# Living in Italy

**Published:** 1881-04

**Authors:** 


### LIVING IN ITALY.

The one great fact that impresses itself on the mind of the traveler in "sunny Italy" is the total absence of proper sanitary arrangements in Italian towns and villages, from the palace to the hovel and room tenement. Italy—the land of sunshine, art and song—is a land of filth and vermin. There are marble palaces, art galleries and blue skies, but neither sewers, drains nor adequate scavenging. Hence, strangers who are tempted to visit the world-renowned cities pay a fearful penalty in risks from fever, and certainly of mosquito stings, as also of punishment from other domestic torments. There is not one Italian city properly sewered, drained and scavenged. The best hotels use cesspools, out of which pass foul gases and putrid fluids, to contaminate both air and water. Proper scavenging implies daily cleaning, not only of public streets and places but of all back streets, lanes, alleys, yards and tenement houses, with a removal of *excreta* and refuse at short intervals, never exceeding one week. As to proper sanitary works, a full supply of pure water is necessary, not merely for display in public fountains, but laid on by appropriate services to every occupied dwelling, however humble. The regulations of a Common Lodging House Act should be enforced in every slum and wretched room-tenement, and all the places unfit for human habitation should be sternly closed and proper accommodation provided. When the improvements herein suggested have been adopted and are continuously carried out, there may be hope for the regeneration of Italy.—*Home Journal.*

### A DELICIOUS MORSEL.

A gentleman traveling in Virginia last summer had occasion to take a stage ride in order to visit the natural bridge. Riding on the seat with the driver he fell into conversation with him, and found that he was an old hunter, who was a veteran in killing deer, bear, and smaller game. Passing a stream, the traveler inquired if it contained fish. "Lots on 'em," was the reply. "What kind?" "Mostly trout," said the driver; "all these mountain streams are full of trout." "They must be fine eating," was the next remark. "Fine eatin'!" exclaimed the driver; "you just go up to the mountain and ketch half a dozen trout about twelve inches long, clean 'em without washin' 'em, rub in some salt, roll 'em in Injin meal, and bake 'em in ashes—good eatin'! why, stranger, they beat ham!"

